# Computed Tomography Features and Clinical Prognostic Characteristics of Hepatoid Adenocarcinoma of the Stomach

**DOI:** 10.3389/fonc.2021.772636

**Published:** 2021-12-09

**Authors:** Wen-peng Huang, Li-ming Li, Jing Li, Jun-hui Yuan, Ping Hou, Chen-chen Liu, Yi-hui Ma, Xiao-nan Liu, Yi-jing Han, Pan Liang, Jian-bo Gao

**Affiliations:** ^1^ Department of Radiology, The First Affiliated Hospital of Zhengzhou University, Zhengzhou, China; ^2^ Department of Radiology, The Affiliated Cancer Hospital of Zhengzhou University (Henan Cancer Hospital), Zhengzhou, China; ^3^ Department of Pathology, The First Affiliated Hospital of Zhengzhou University, Zhengzhou, China

**Keywords:** hepatoid adenocarcinoma, stomach, tomography, x-ray computed, diagnosis, adenocarcinoma

## Abstract

**Purpose:**

Hepatoid adenocarcinoma of the stomach (HAS) is a highly malignant and aggressive tumor. The purpose of this study was to describe the clinical, computed tomography (CT), and prognostic features of HAS to increase the awareness of this entity and determine its distinguishing features from non-HAS tumors.

**Methods:**

The CT features and clinical data of 47 patients in our hospital with pathologically documented HAS were retrospectively analyzed, and the relevant differences between pure HAS (pHAS) and mixed HAS (mHAS) were determined. In addition, 141 patients with non-HAS tumors in the same T stage in the same period were selected as the control group. The data were compared between the two groups, and factors affecting the prognosis of HAS were analyzed. In addition, we included 9 patients with HAS and 27 patients with non-HAS tumors from another center for external validation.

**Results:**

The patients in the HAS group were predominantly men (n = 33), and the tumor location was mostly the cardia or fundus (n = 27). Between the HAS and non-HAS groups, there were observed differences in terms of: sex, serum alpha-fetoprotein (AFP), carbohydrate antigen (CA)-125, and CA-724 levels; longest tumor diameter; degree of differentiation; vascular invasion; N stage, M stage, and tumor-node-metastasis (TNM) stage; thickest tumor diameter; plain CT attenuation; arterial-phase CT attenuation; CT attenuation between the venous and arterial phases; enhancement modes; and degrees of enhancement (all *P* < 0.05). In the data from another center for external validation, there were observed differences in terms of: age, degree of differentiation, vascular invasion, thickest tumor diameter, the ratio of arterial CT attenuation to CT attenuation of the abdominal aorta at the same level (R_A_), CT attenuation difference between the venous phase and arterial phase (HUv-a) (all *P* < 0.05). The results of the multivariate analysis revealed that the independent factors for differentiation were serum AFP level (*P =* 0.001), M stage (*P =* 0.038), and tumor enhancement on CT (*P =* 0.014). Among patients in the HAS group, 72.34% had pHAS and 27.66% had mHAS. The thickest tumor diameter and the longest short diameter of the metastatic lymph nodes of the mHAS group were on average 6.39 cm and 1.45 cm, respectively, which were larger than those in the pHAS group. The median progression-free survival time was 18.25 months in the HAS group, which was shorter than that in the non-HAS group (72.96 months; *P =* 0.001). The median overall survival time in the HAS group was 24.80 months, which was shorter than that in the non-HAS group (67.96 months; *P =* 0.001). The factors affecting the prognosis of HAS were M stage (*P =* 0.001), overall TNM stage (*P =* 0.048), presence of vascular cancer emboli (*P =* 0.040), and pHAS type (*P =* 0.046). Multifactorial analysis revealed that M stage (*P =* 0.027) and pHAS type (*P =* 0.009) were independent risk factors affecting the prognosis of HAS.

**Conclusion:**

Although HAS is a rare clinical entity, it should be considered in the differential diagnosis of gastric tumors. Patients with HAS often have advanced-stage disease at presentation and a worse prognosis than patients with non-HAS tumors. CT findings, combined with laboratory results, can support the diagnosis of HAS. However, the final diagnosis needs to be confirmed with a histopathologic examination. If the postoperative pathologic findings reveal the mHAS type, a rapid clinical intervention and a detailed follow-up with CT are essential.

## Introduction

Hepatoid adenocarcinoma (HAC) is a clinically rare and specific type of malignancy that occurs in tissues and organs other than the liver (1). Histologically, the tumor cells of HAC are polygonal and proliferate in a solid or trabecular fashion, showing many hepatocellular carcinoma (HCC)-like features such as intracytoplasmic glycogen granules, centripetal annular lamellar vesicles, and proliferation in capillary bile ducts (2–4). HAC can occur in the stomach, esophagus, duodenum, jejunum, colon, peritoneum, pancreas, lungs, ovaries, gallbladder, and uterus, with the stomach being the most common site of occurrence (1, 2). Hepatoid adenocarcinoma of the stomach (HAS) was first proposed in 1985 by Ishikura et al. (5). It accounts for approximately 0.17%–15% of all gastric cancers, with an estimated annual incidence of 0.58–0.83 cases per 1 million individuals (6–9), and shows atypical clinical symptoms that are characterized by high serum AFP (1). Previous reports focused on pathologic and clinical manifestations, and only a few reports involving a small number of cases have reported the computed tomography (CT) findings of HAS (10–12). These previous reports described only the imaging presentation of HAS and did not compare and analyze it with common gastric adenocarcinoma in terms of CT presentation and prognosis. Because HAS is characterized by aggressiveness, poor prognosis, and high metastasis potential, there is a clinical need to differentiate it from common gastric adenocarcinoma; however, the clinical presentation of HAS is similar to that of non-HAS tumors (4, 13). Clinicians should improve their understanding of HAS and improve their diagnosis accuracy.

In this study, we aimed to retrospectively summarize and analyze the clinical data and CT imaging findings of 47 pathologically confirmed HAS cases at our institution and determine the features that distinguish HAS from common gastric adenocarcinoma. In addition, we analyzed the CT imaging differences between pure HAS (pHAS) and mixed HAS (mHAS). To our knowledge, our study represents the largest series investigating HAS in relation to CT imaging findings, to date.

## Materials and Methods

The study protocol was approved by the Medical Ethics Committee of Zhengzhou University. Informed consent was obtained from all patients.

### Patient Selection

**Figure 1:**
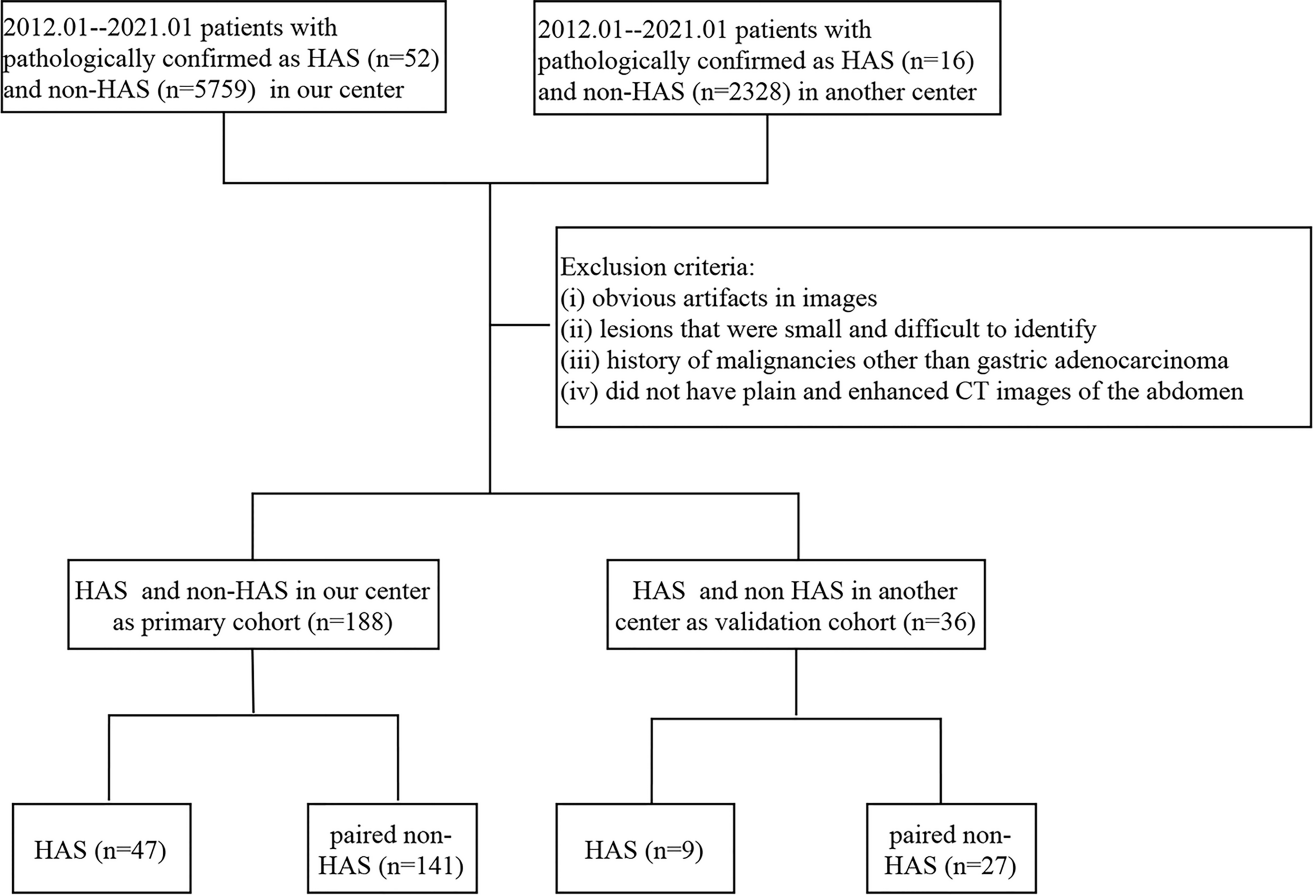
The patient enrollment workflow.

We retrospectively collected the clinical data of patients with pathologically confirmed HAS at our hospital and the Henan Cancer Hospital between February 2013 and February 2021, including demographic features, laboratory findings, histopathologic data, and CT imaging data. Patients with HAS were included if they: (i) were not receiving other antitumor treatments, such as neoadjuvant chemotherapy, before their surgery or puncture tests; (ii) had plain and enhanced CT images of the abdomen obtained 1 week before their surgery and puncture tests; (iii) did not have serious underlying diseases or organ insufficiency; and (iv) had a complete clinical follow-up. The exclusion criteria were as follows: (i) obvious artifacts in images, (ii) lesions that were small and difficult to identify, and (iii) history of malignancies other than gastric adenocarcinoma. In patients with unresectable metastases, staging was based on preoperative CT images or intraoperative exploration. A total of 47 patients with HAS were included in our hospital, 9 patients with HAS were included in the Henan Cancer Hospital; we randomly selected non-HAS patients with pathologically confirmed gastric adenocarcinoma in the same operative month from the Picture Archiving and Communication Systems (PACS) system of our hospital. We selected non-HAS patients with the same T-stages as in the HAS group and paired them in a 1:3 ratio to form the control group (non-HAS group). The inclusion and exclusion criteria were the same as those for the HAS group. All clinical data and the prognosis of patients were compared between the two groups. The data from our hospital was used as the primary cohort, and the data from the Henan Cancer Hospital was used for external validation. The flow chart of inclusion and exclusion is shown in **Figure 1**.

### CT Image Acquisition

The CT images were acquired with a 64-row CT scanner (Discovery CT 750HD, GE Healthcare, Waukesha, WI, United States) and a 256-row CT scanner (Revolution CT; GE Healthcare, Waukesha, WI, United States). All patients fasted overnight, drank 800–1000 ml of water at an appropriate temperature in order to expand their stomach, and performed breath-holding exercises before their CT examination. A total of 20 mg of scopolamine (Hangzhou Minsheng Pharmaceutical Group Co., Ltd. Specifications: 10 mg/ml) was administered intramuscularly to reduce gastrointestinal peristalsis 15–20 min prior to the CT scans (14). Each patient took the supine position, ranging from the transverse septum to the inferior margin of the pubic symphysis. Conventional axial scanning was performed before and after an intravenous (i.v.) injection of nonionic iohexol (iopromide, 370 mg/mL, GE Medical Systems, 1.5 mL/kg and 3 mL/s) and administration of 20 mL of saline through a dual-head pump injector (Medrad, Warrendale, PA, United States). The same scanning protocol was used for both CT machines. The scanning parameters were as follows: tube voltage, 120 kV; automatic mA (100–650 mA) technology was used for the tube current; noise index, 10.0; pitch of screw, 1.375:1; angular velocity, 0.5 s; field of view (FOV), 500 mm; matrix, 512 × 512 mm; and section thickness, 1.25 mm.

Using the low-dose trigger technique, when the descending aorta reached 100 HU after injection of the contrast medium, arterial phase images were collected 10 seconds later, and venous phase images were collected at intervals of 30 seconds. The coronal and sagittal images were reconstructed with the multi-planar reconstruction (MPR) technique. The slice thickness was 3 mm and the interval between slices was 3 mm. All CT images were transferred to a post-processing workstation (Advantage Windows 4.6; GE Medical Systems, Chicago, IL, United States). We used the software on the post-processing workstation to match the plain, arterial, and venous images at the same level of measurement whenever possible to minimize errors.

### Image Analysis

Two experienced radiologists (L.L. and P.L., with 8 and 12 years of experience with abdominal CT, respectively) analyzed the CT images. The radiologists were blinded to the patients’ clinical information. The CT attenuation were measured with regions of interests (ROIs) that were drawn manually, each with a diameter of about 10 mm^2^–20 mm^2^, while carefully avoiding vessels as well as necrotic and artifactual areas. The ROIs were measured at least three times in the largest cross-section of the tumor axial slices and the average values were taken. The evaluated parameters included tumor location, thickest diameter, margins, density, Borrmann type, CT attenuation (in the plain, arterial, and venous phases), CT attenuation differences between the arterial phase and plain phase, CT attenuation differences between the venous phase and arterial phase, and ratio of enhanced duplex CT attenuation to CT attenuation of the abdominal aorta at the same level. The mode and degree of enhancement of the lesion, presence of cystic necrosis within the lesion, and metastatic invasion to adjacent organs were analyzed. When diagnostic disagreements arose, a third radiologist (J.G., with 30 years of experience with abdominal CT) made a final decision. The degree of enhancement of the tumor on dynamic CT imaging was assessed through measurements of Hounsfield unit (HU) attenuation, in which “obvious enhancement” was defined as ≥ 40 HU, “moderate enhancement” as ≥ 20 HU, and “mild enhancement” as < 20 HU (15).

### Pathologic Evaluation

The specimens were fully stretched, fixed, and soaked in a 3.7% formaldehyde solution for 24 h. The specimens were routinely dehydrated, embedded in paraffin, cut into 4-µm-thick sections, and stained with hematoxylin and eosin. All biopsy specimens were analyzed (X.L. and Y.M., with 3 and 15 years of experience with pathology, respectively). Immunohistochemical staining was performed using a Roche BenchMark XT automatic immunohistochemical detector. The antibodies used in this study included alpha-fetoprotein (AFP), hepatocyte, glypican, and antigen KI67 (Ki-67). All antibodies were purchased from Dako (Glostrup, Denmark).

### Follow-Up

Of the 47 patients with HAS, 36 underwent surgical resection (23 underwent postoperative adjuvant chemotherapy), eight underwent adjuvant chemotherapy only, two did not receive active treatment, and 1 was intraoperatively unresectable and underwent gastric vascular dissection and gastrojejunostomy only. Of the 141 patients with non-HAS, 123 underwent surgical resection (89 underwent postoperative adjuvant chemotherapy), 15 received adjuvant chemotherapy only, and 3 did not receive active treatment. The patients in both groups were followed up *via* outpatient examinations and telephone interviews. After the completion of surgical treatment and discharge, the patients were followed up every 3 months for the first year and every 3–6 months for the second year. From 3 years after discharge, follow-up was performed every 6 months until June 2021. Patient death was considered a follow-up end point. Progression-free survival (PFS) time was the period from the date of diagnosis to the disease progression, or the last follow-up in the case of no progression. The overall survival (OS) time was defined as the period from the date of diagnosis to death from any cause.

### Statistical Analysis

SPSS software (version 23.0; SPSS Inc., Chicago, IL, USA) was used to process and analyze the data. Independent-samples *t*-tests or Mann–Whitney *U* tests were used for comparisons of continuous variables, and chi-square tests or Fisher’s exact tests were used for comparisons of categorical variables between groups. The Kaplan-Meier method was used for survival analysis, and the Long-rank test was used for comparison of survival curves. Because of the small sample size of the validation group, some of the features were chosen to be classified and combined between the two groups before comparing them. Variables that were significant in the univariate analysis were included in the multifactorial logistic regression analysis (backward: conditional). Continuous variables in the multifactorial analysis were converted to categorical variables according to the median values. Differences were considered statistically significant at *P* < 0.05.

## Results

### Comparison Between the HAS and Non-HAS Groups

**Figure 2:**
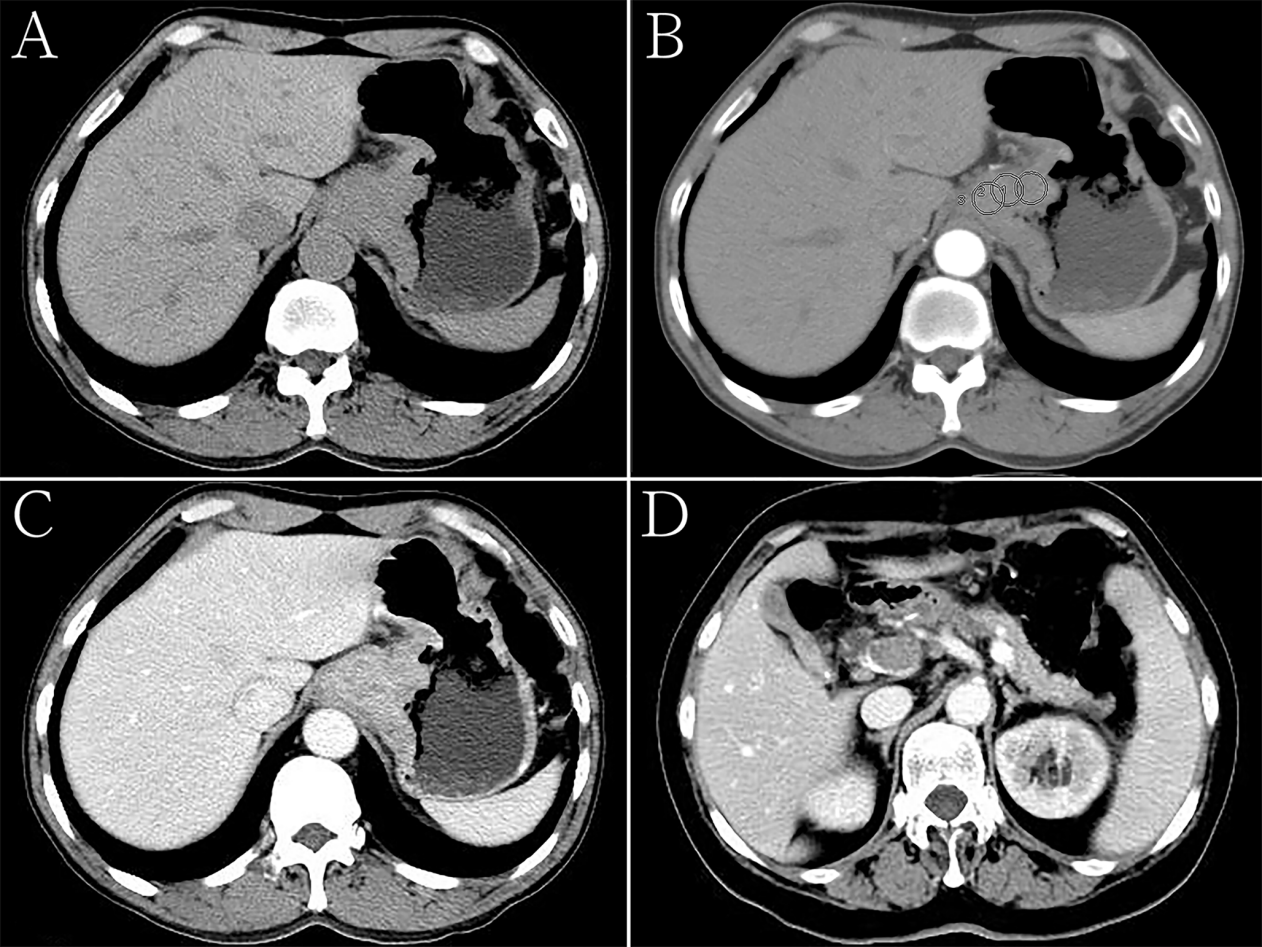
Hepatoid adenocarcinoma of the stomach in a 60-year-old woman. **(A)** Unenhanced computed tomography (CT) image of the stomach showing an intraluminal mass of homogeneous attenuation, with an irregular surface, in the cardia and fundus. The CT attenuation was approximately 52 Hounsfield units (HU). **(B–D)** Contrast-enhanced CT image showing moderate inhomogeneous enhancement of the mass, with the peak value observed in the portal phase. The thickest diameter of the mass was 4.7 cm. Ulceration was seen on the surface of the lesion. The Borrmann classification was type III. The CT attenuation in the arterial and venous phases were approximately 69 and 89 HU, respectively. Low-density tumor thrombi can be seen in the portal vein. **(B)** Arterial phase of the contrast-enhanced image, the CT attenuation were measured with regions of interests (ROIs) that were drawn manually, each with a diameter of about 15 mm^2^, while carefully avoiding vessels as well as necrotic and artifactual areas. The ROIs were measured at least three times and the average values were taken. **(C, D)** Portal phase of the contrast-enhanced image.

**Figure 3:**
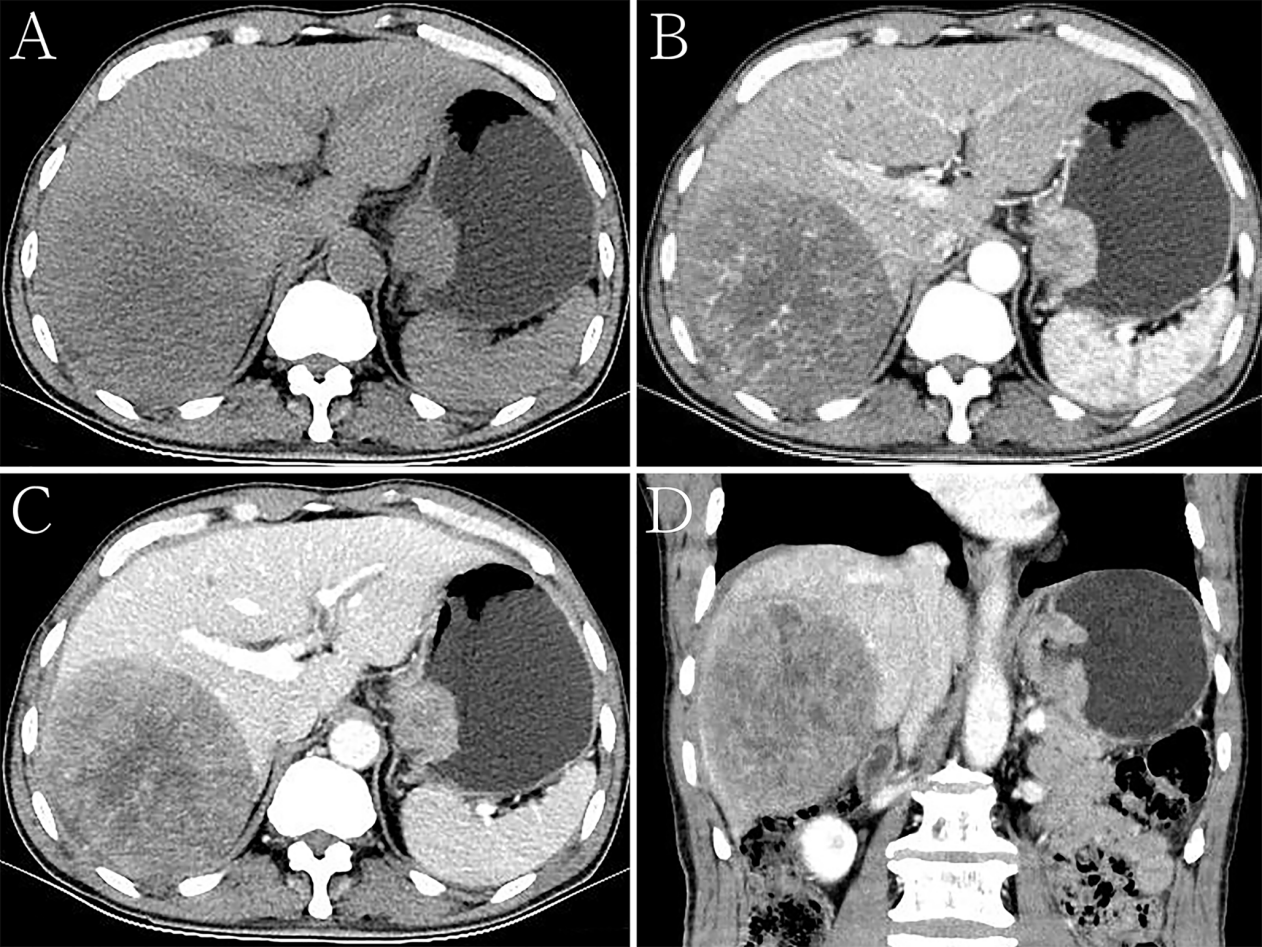
Hepatoid adenocarcinoma of the stomach with liver metastases in a 68-year-old man. **(A)** Unenhanced computed tomography (CT) image of the stomach showing an intraluminal mass of homogeneous attenuation, with a smooth surface, in the cardia and fundus. The CT attenuation was approximately 46 Hounsfield units (HU). A round low-density area was found in the liver. **(B–D)** Contrast-enhanced CT images showing obvious inhomogeneous enhancement of the mass, with the peak value of the tumor observed in the arterial phase. The thickest diameter of the mass was 3.4 cm. Ulceration was seen on the surface of the lesion on the multiplanar reconstruction (MPR) image. The Borrmann classification was type III. The CT attenuation in the arterial and venous phases were approximately 69 and 89 HU, respectively. The degree of enhancement of intrahepatic metastases was similar to that of the primary gastric lesions. **(B)** Arterial phase of the contrast-enhanced image. **(C)** Portal phase of the contrast-enhanced image. **(D)** Portal phase of the contrast-enhanced coronal image.

**Figure 4:**
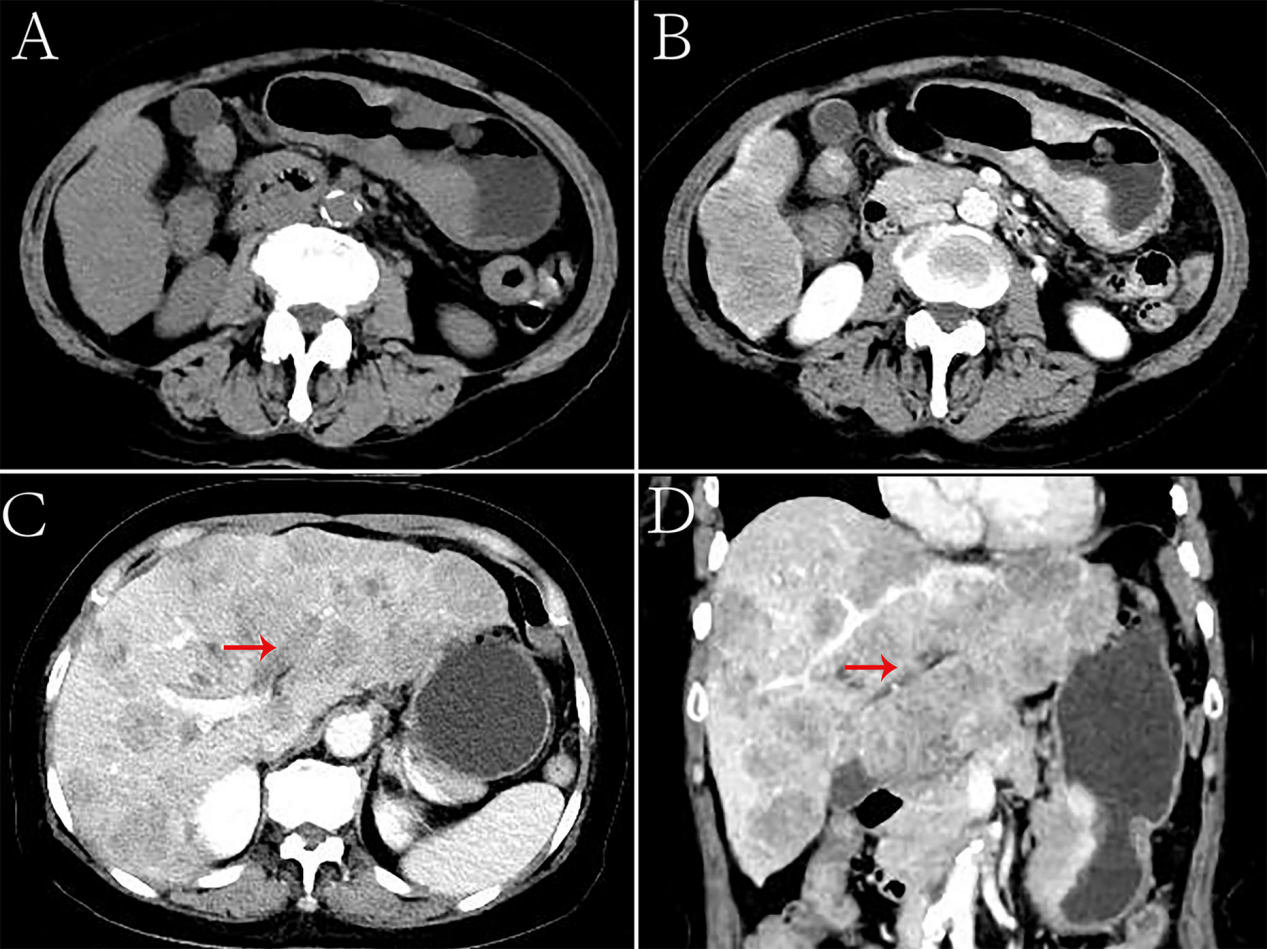
Hepatoid adenocarcinoma of the stomach with embolization of the left branch of the portal vein in a 70-year-old woman. **(A)** Unenhanced computed tomography (CT) image of the stomach showing an intraluminal mass of inhomogeneous attenuation, with an undercooling boundary, in the cardia and fundus. The CT attenuation was approximately 41 Hounsfield units (HU). Multiple nodular low-density metastases of different sizes were found in the liver. **(B–D)** Contrast-enhanced CT image showing obvious inhomogeneous enhancement of the mass. The thickest diameter of the mass was 3.3 cm. The Borrmann classification was type III. The arterial and venous phases showed a similar CT attenuation (approximately 91 HU). Intrahepatic metastases showed mild to moderate enhancement on enhanced CT. The left branch of the portal vein had no obvious changes, and low-density emboli could be seen in the lumen. **(C)** Portal phase of the contrast-enhanced image. **(D)** Portal phase of the contrast-enhanced coronal image.

**Figure 5:**
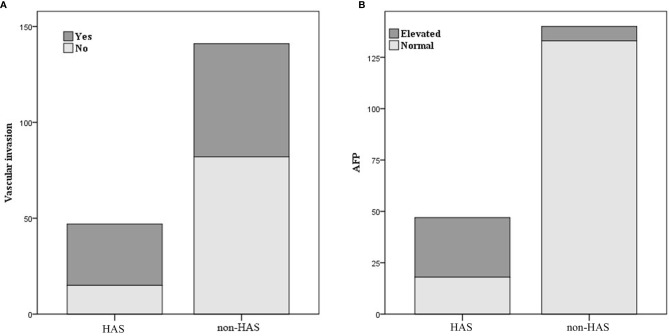
Comparison of vascular invasion **(A)** and alpha-fetoprotein (AFP) level **(B)** between hepatoid adenocarcinoma of the stomach (HAS) and non-HAS cases.

The HAS group included 33 men and 14 women with a mean age of 62.43 ± 7.36 years. The clinical manifestations in the HAS group were mostly nonspecific symptoms such as abdominal pain, abdominal distension, and abdominal discomfort. A total of 27 cases were affected in the cardia-fundus, and the longest tumor diameter was 6.00 cm on average. Between the HAS and non-HAS groups, there were statistically significant differences in terms of sex; serum AFP, carbohydrate antigen (CA)-125, and CA-724 levels; and longest tumor diameters (all *P* < 0.05; **Table 1**). In the HAS group, 25 cases had a type III Borrmann classification, and the thickest diameter of the tumor was 1.0–5.0 cm (median, 2.5 cm), which was larger than that in the non-HAS group (median, 1.7 cm; *P =* 0.001). The plain CT attenuation and arterial phase CT attenuation values were 42.38 and 77.11 HU in the HAS group, respectively, which were higher than those seen in the non-HAS group (*P =* 0.040 and 0.022, respectively). The mean CT attenuation difference between the venous phase and arterial phase in the HAS group was 6.32 HU, which was lower than that in the non-HAS group (16.23 HU; *P =* 0.001). In the HAS group, the reinforcement was mostly persistent (n=19) and the degree of enhancement was mostly mild to moderate (n=29), with statistically significant differences compared to the non-HAS group (*P =* 0.001 and 0.049, respectively). Patients in both the HAS and non-HAS groups showed a single lesion, and there were no statistically significant differences in terms of the groups’ venous-phase CT attenuation, ratio of enhanced duplex CT attenuation to the CT attenuation of the abdominal aorta at the same level, CT attenuation difference between the arterial phase and plain phase, and uniformity of enhancement (*P* > 0.05; **Table 2**, **Figures 2**–**5**). In the data from another center for external validation, there were observed differences in terms of: age, degree of differentiation, vascular invasion, thickest tumor diameter, the ratio of arterial CT attenuation to CT attenuation of the abdominal aorta at the same level (RA), CT attenuation difference between the venous phase and arterial phase (HUv-a) (all *P* < 0.05; **Supplementary Table S1**).

**Table 1:** Comparison of clinical pathology information between HAS and non-HAS n = 188.

		HAS (n = 47)	non-HAS (n = 141)	*P* value
Sex	Male	33	118	0.044^*^
	Female	14	23	
Age (years)		62.43 ± 7.36	60.35 ± 11.38	0.334
Serum AFP (n=187)	Elevated	29	7	0.001^*^
	Normal	18	133	
Serum CA125 (n=174)	Elevated	12	13	0.011^*^
	Normal	35	114	
Serum CA724 (n=158)	Elevated	11	18	0.024^*^
	Normal	24	105	
Serum CA199 (n=187)	Elevated	7	20	0.918
	Normal	40	120	
Serum CEA (n=186)	Elevated	13	28	0.241
	Normal	33	112	
Location	Antrum	14	31	0.425
	Body	4	24	
	Cardia and Fundus	27	80	
	Involvement of 2 or more sites	2	6	
Longest short diameter of metastatic lymph node (cm)		1.50(1.00, 2.38)	1.15(1.00, 1.58)	0.093
Longest diameter of tumor (cm)		6.00(4.00, 8.00)	4.50(3.50, 6.00)	0.001^*^
Main symptoms	Abdominal pain/bloating/abdominal discomfort	27	98	0.087
	Acid reflux, heartburn/choking sensation when eating	10	32	
	Vomiting of blood/black stool	7	8	
	No significant symptoms (physical examination)	3	3	
Degree of differentiation	Low	43	88	0.001^*^
	Middle-high	4	53	
Neural encroachment	Yes	34	84	0.117
	No	13	57	
Vascular invasion	Yes	32	59	0.002^*^
	No	15	82	
T stage	1	2	6	1.000
	2	14	42	
	3	17	51	
	4	14	42	
N stage	0	11	57	0.001^*^
	1	2	27	
	2	20	21	
	3	14	36	
M stage	0	34	139	0.001^*^
	1	13	2	
TNM stage	1	4	34	0.001^*^
	2	13	41	
	3	17	64	
	4	13	2	

HAS, Hepatoid adenocarcinoma of the stomach; AFP, alpha-fetoprotein (normal range 0–10 ng/mL); CA, carbohydrate antigen, CA199 (normal range 0.01–37 U/mL), CA724 (normal range 0–6.9 U/mL), CA125 (normal range 0.01–35 U/mL); CEA, carcinoembryonic antigen (normal range 0–5 ng/mL); *Statistically significant level: P < 0.05.

**Table 2:** Comparison of CT features between HAS and non-HAS (n=188).

		HAS (n=47)	non-HAS (n=141)	*P* value
Borrmann type (n=180)	I	0	3	0.032^*^
	II	8	38	
	III	25	80	
	IV	12	14	
Thickest diameter (cm)		25(19, 38)	17(14, 23)	0.001^*^
Plain CT attenuation (HU)		42.38 ± 6.42	39.77 ± 7.82	0.040^*^
Arterial CT attenuation (HU)		77.11 ± 17.26	70.15 ± 18.13	0.022^*^
Venous CT attenuation (HU)		83.43 ± 15.13	86.38 ± 19.33	0.342
R_A_		0.27(0.23, 0.33)	0.26(0.21, 0.31)	0.118
R_V_		0.580 ± 0.095	0.620 ± 0.130	0.104
HU_A-P_		34(21, 49)	27(17.5, 43.5)	0.136
HU_V-A_		6.32 ± 13.70	16.23 ± 17.13	0.001^*^
Degree of enhancement	Obvious enhancement	18	83	0.049^*^
	Moderate enhancement	21	41	
	Mild enhancement	8	17	
Mode of enhancement	Continuous reinforcement	19	31	0.001^*^
	Progressive reinforcement	20	104	
	Ascending and then descending type of reinforcement	8	6	

HAS, Hepatoid adenocarcinoma of the stomach; HU, Hounsfield unit; R_A_, the ratio of arterial CT attenuation to CT attenuation of the abdominal aorta at the same level; R_V_, the ratio of venous CT attenuation to CT attenuation of the abdominal aorta at the same level; HU_A-P_, CT attenuation difference between the arterial phase and plain phase; HU_V-A_, CT attenuation difference between the venous phase and arterial phase; *Statistically significant level: P < 0.05.

**Figure 6:**
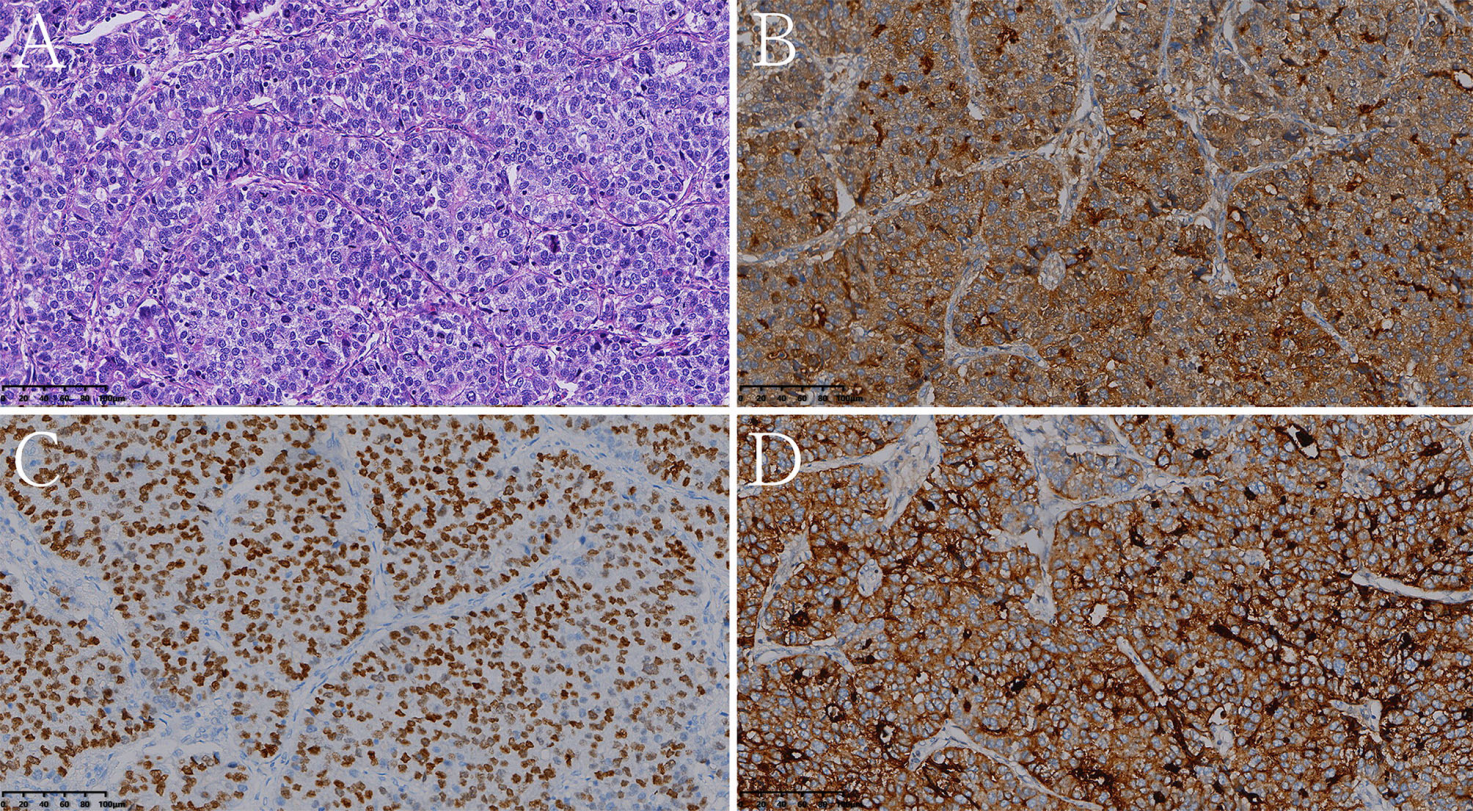
Histologic and immunohistochemical features of pure hepatoid adenocarcinoma of the stomach (pHAS). **(A)** Hematoxylin and eosin (HE) staining. On immunohistochemistry, the tumor cells were positive for AFP **(B)**, SALL4 **(C)**, glypican 3 **(D)** (magnification **(A–D)** ×200).

**Figure 7:**
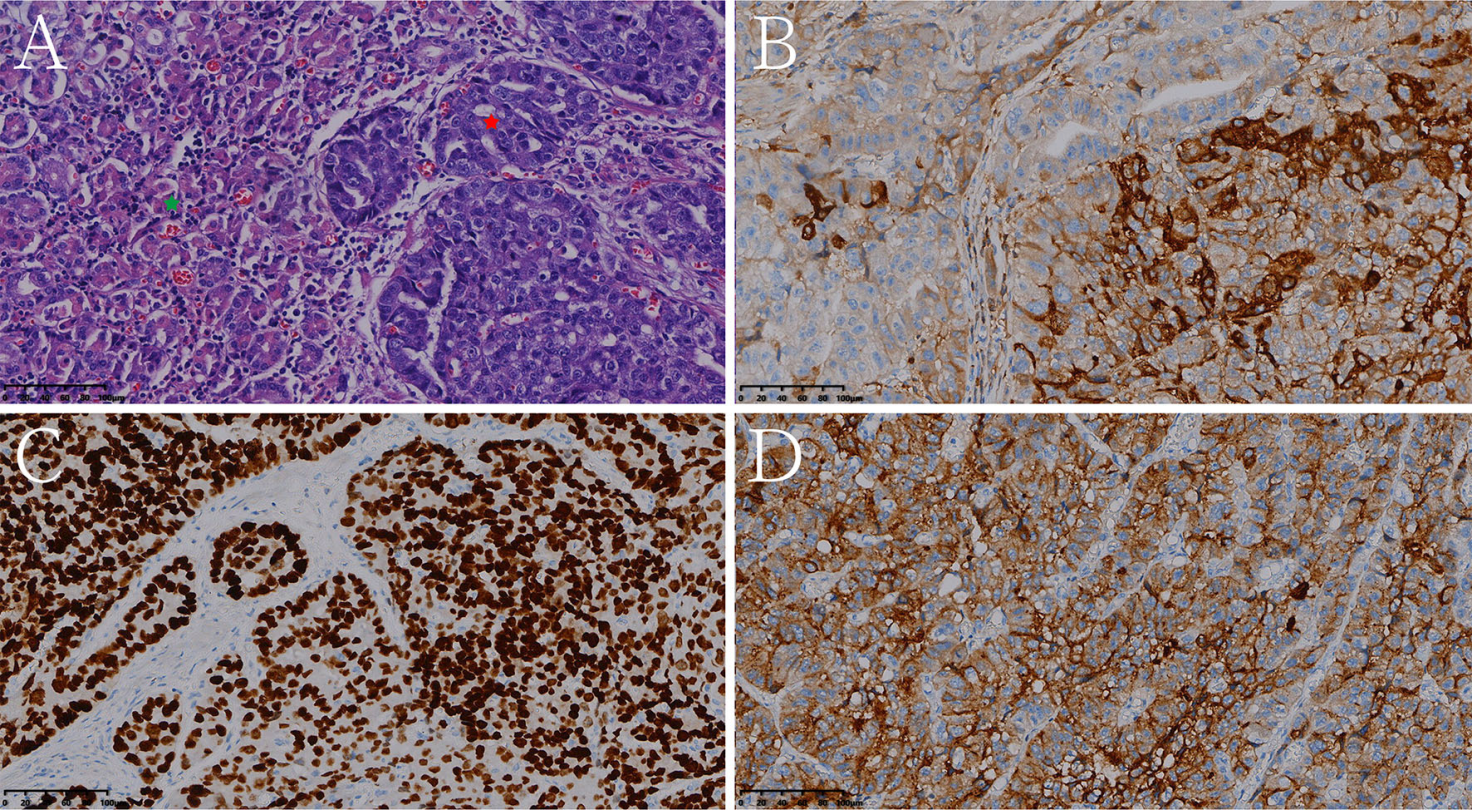
Histologic and immunohistochemical features of mixed hepatoid adenocarcinoma of the stomach (partly enteroblastoma type adenocarcinoma). **(A)** Hematoxylin and eosin (HE) staining (the red pentagram represents the HAS component, the green pentagram represents the enteroblastoma type adenocarcinoma component). On immunohistochemistry, the tumor cells were positive for AFP **(B)**, SALL4 **(C)**, glypican 3 **(D)** (magnification **(A–D)** ×200).

**Table 1** shows that patients in the HAS group were more likely to have liver metastasis (n=13) and vascular invasion (n=32) than patients in the non-HAS group, despite the comparable T stages between the two groups. The HAS and non-HAS groups showed statistically significant differences in terms of their degree of differentiation, N stage, M stage, overall TNM stage, and vascular invasion (*P* < 0.05). In the immunohistochemical assays, 80.85% of patients in the HAS group showed positive expression of AFP (38/47). The AFP-positive areas were mainly distributed in the hepatocyte-like differentiated areas, and 53.19% of the hepatocyte-like differentiated areas showed diffuse positive or focal positive expression of hepatocytes (25/47) and 65.96% showed positive expression of glypican (31/47). The Ki-67 positivity index ranged from 30% to 90%, and 87.23% had a high labeling index (> 50%) for Ki-67 (**Figures 6**, **7**). The results of the multifactorial analysis revealed that the independent factors for differentiating HAS from non-HAS were serum AFP level, M stage, and tumor enhancement on CT (**Table 3**).

**Table 3:** Multivariate analysis of clinicopathological characteristics and CT features of the HAS and non-HAS.

Variables	Multivariate analysis			
	β	Wald	95% CI	*P* value
Intercept	-3.962	1.644		0.200
Serum AFP	4.811	27.834	20.572, 734.262	0.001
M stage	-2.914	4.312	0.003, 0.849	0.038
Degree of enhancement	-1.249	5.999	0.106, 0.079	0.014

### Comparison Between the Pure HAS (pHAS) and Mixed HAS (mHAS) Groups

In the HAS group, 34 patients had pHAS, with areas of hepatomatous differentiation and adenocarcinoma differentiation seen microscopically, and the histologic features of the hepatocellular differentiation zone were similar to those of HCC. The tumor cells were large in size, arranged in solid nests, sheets, or strips, had abundant and eosinophilic cytoplasms, partially translucent cytoplasms, obvious nucleoli, and many schizogenous bodies; they also had sinusoidal vascular channels that were separating the interstitium (16, 17). Adenocarcinoma differentiation areas were mostly located on the surfaces of the tissues, whereas liver-like differentiation areas were mostly located in the deep layer, which is where the two types of tissue cells fuse and migrate into each other. Meanwhile, 13 patients had mHAS (i.e., other tumor components were seen in the lesions besides hepatocellular and adenocarcinoma components, including enteroblastoma in six cases, indolent cell carcinoma in three cases, mucinous adenocarcinoma in two cases, and neuroendocrine differentiation in two cases). No statistically significant difference was observed between the pHAS and mHAS groups in terms of pathologic features (**Supplementary Table S2**). The longest short diameter of metastatic lymph nodes in the pHAS group was 1.65 (0.78–3.30) cm, which was greater than that in the mHAS group (1.45 [1.0–2.0] cm; *P =* 0.001). The thickest diameter of the tumor in the pHAS group was 2.90 cm, which was greater than that in the mHAS group (2.25 cm; *P =* 0.049; **Supplementary Table S3**).

### Prognostic Assessment

**Figure 8:**
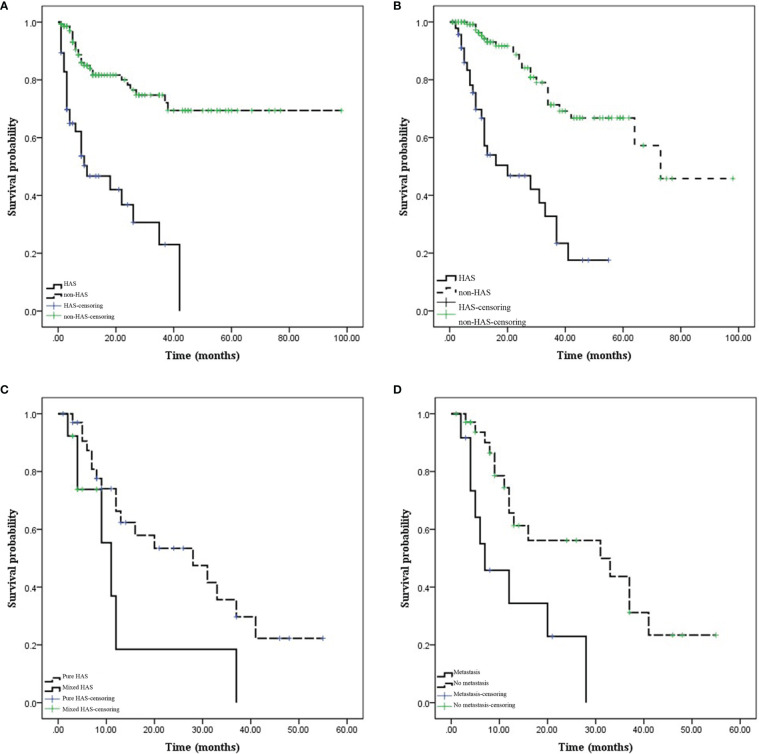
Comparison of prognosis between hepatoid adenocarcinoma of the stomach (HAS) and non-HAS cases. **(A, B)** Survival curves (progression-free survival [PFS] and overall survival [OS]) of the HAS and non-HAS groups. The PFS and OS were significantly shorter in the HAS group than in the non-HAS group. **(C)** Effects of the pure HAS (pHAS) and mixed HAS (mHAS) types on the OS of HAS. **(D)** Effect of M stage on the OS of HAS. The OS was shorter for mHAS and distant metastatic HAS.

Prognosis was compared between patients with pHAS and those with non-HAS. Patients in the HAS group were followed up for 1–55 months, during which 28 patients had disease progression and 25 patients died. Patients in the non-HAS group were followed up for 1–98 months, during which 28 patients had disease progression and 24 patients died. The median PFS time was 18.25 months in the HAS group, which was shorter than that in the non-HAS group (72.96 months; *P =* 0.001). The median overall survival (OS) time in the HAS group was 24.80 months, which was shorter than that in the non-HAS group (67.96 months; *P =* 0.001; **Figure 8**).

Factors influencing prognosis in patients with HAS were analyzed. Univariate survival analysis identified M stage (*P =* 0.001), TNM stage (*P =* 0.049), presence of vascular carcinoma thrombi (*P =* 0.040), and pHAS type (*P =* 0.046) as factors influencing the prognosis of HAS. The results of the multifactorial analysis revealed that the independent risk factors affecting the prognosis of patients included M stage (*P =* 0.027) and pHAS type (*P =* 0.009; **Table 4**).

**Table 4:** Univariate and multivariate analysis using stepwise variable selection of the clinicopathological characteristics and CT features with overall survival.

Variables		Univariate analysis			Multivariate analysis		
		HR	95% CI	*P* value	HR	95% CI	*P* value
Demographic data	Sex	1.214	0.056, 2.910	0.664			
	Age	1.607	0.718, 3.595	0.248			
	Main symptoms	0.680	0.566, 4.985	0.350			
	Location	1.097	0.492, 2.447	0.820			
	Longest diameter of tumor	1.798	0.073, 4.183	0.173			
	Longest short diameter of metastatic lymph node	0.792	0.032, 2.078	0.635			
	Serum AFP	0.481	0.200, 1.158	0.103			
	Serum CA125	0.457	0.199, 1.052	0.066			
	Serum CA199	0.451	0.169, 1.207	0.113			
	Serum CA724	0.513	0.195, 1.351	0.177			
	CEA	1.249	0.536, 2.909	0.606			
Pathology	Neural encroachment	0.304	0.088, 1.504	0.061			
	Vascular invasion	0.355	0.132, 0.955	0.040			
	Degree of differentiation	0.923	0.274, 3.109	0.897			
	T stage	1.394	0.575, 3.383	0.462			
	N stage	3.984	0.932, 17.026	0.062			
	M stage	4.942	2.026, 12.053	0.001	3.380	1.145, 9.976	0.027
	TNM stage	2.549	1.002, 6.482	0.049			
	pHAS type	2.460	0.999, 6.053	0.046	3.794	1.399, 10.289	0.009
CT	Borrmann type	0.693	0.892, 8.128	0.079			
	Thickest diameter	1.384	0.619, 3.094	0.429			
	Degree of enhancement	0.769	0.348, 1.700	0.516			
	Mode of enhancement	0.776	0.349, 1.727	0.053			
	Plain CT attenuation	0.455	0.198, 1.044	0.063			
	Arterial CT attenuation	1.672	0.752, 3.719	0.208			
	Venous CT attenuation	1.652	0.727, 3.752	0.230			
	R_A_	1.008	0.456, 2.228	0.984			
	R_V_	1.525	0.655, 3.549	0.328			
	HU_A-P_	2.042	0.908, 4.590	0.084			
	HU_V-A_	0.740	0.336, 1.631	0.456			

## Discussion

Between the HAS and non-HAS groups, there were observed differences in terms of: sex, serum alpha-fetoprotein (AFP), carbohydrate antigen (CA)-125, and CA-724 levels; longest tumor diameter; degree of differentiation; vascular invasion; N stage, M stage, and tumor-node-metastasis (TNM) stage; thickest tumor diameter; plain CT attenuation; arterial-phase CT attenuation; CT attenuation between the venous and arterial phases; enhancement modes; and degrees of enhancement. The results of the multifactorial analysis revealed that the independent factors for the identification of HAS were serum AFP level, M stage, and degree of tumor enhancement on CT. In our study, 61.70% of patients with HAS had significantly elevated serum AFP levels, which may be a result of the dedifferentiation of cancer cells into hepatoid adenocarcinoma progenitor cells. Diseases such as HCC, cirrhosis, yolk cystic tumor, and teratoma cannot be considered alone in patients with elevated serum AFP levels. In our study, one patient with HAS was found to have an elevated serum AFP level and hepatic occupancy on imaging; however, digestive tract examination was not performed. Therefore, only interventions for liver metastases were provided on the basis of the initial diagnosis. If liver lesions are found with elevated AFP levels in patients with no previous history of liver disease, the possibility of HAS should be considered. Moreover, gastroscopy should be performed, if necessary, to identify the primary tumor in the gastrointestinal tract and avoid misdiagnosis or missed diagnosis. Preoperative testing and postoperative dynamic observation of serum AFP levels are beneficial for the early diagnosis of HAS and the evaluation of treatment efficacy, and their inclusion in routine screening for gastric cancer should be considered (7). However, notably, some cases of HAS do not produce AFP (13, 18).

HAS is strongly angiophilic, with vascular infiltration of cancer cells found using microscopy in approximately 63% to 83% of cases (7). In our study, 68.09% of patients in the HAS group developed vascular cancer embolism, which was higher than the percentage in the non-HAS group (18, 19). HAS is believed to exhibit a high degree of aggressiveness associated with its production of α1-antitrypsin and α1-antichymotrypsin, which enhances its aggressiveness and affects its immunosuppressive properties (4, 20).

Although the results show that no independent CT features can identify different types of HAS, it is still important to analyze the CT features. On the one hand, CT can visualize the infiltration depth, extent and morphology of HAS, intensification characteristics, judge the invasion of surrounding organs, detect lymph nodes and distant metastases, and observe volume changes after chemotherapy, and is the preferred imaging examination method for gastric cancer diagnosis, staging, efficacy evaluation and follow-up observation recommended by domestic and international gastric cancer treatment guidelines or norms (21–23). On the other hand, analyzing the characteristics of the different types of HAS can help to understand HAS more comprehensively in order to identify it from non-HAS. When CT features are not particularly suggestive of HAS, we should consider the possibility of mHAS to better aid clinical management. In previous studies, CT findings such as the longest short diameter of the tumor, ratio of lesion attenuation to aortic CT attenuation, CT attenuation differences between the arterial and venous phases, and ratio of the number of suspicious lymph nodes on CT to the number of histologically proven metastatic lymph nodes were important predictors for distinguishing HAS from other gastric cancers (10, 24, 25). Our own hospital’s results revealed that the thickest diameter of the tumor, plain CT attenuation, CT attenuation in the arterial phase, CT attenuation differences between the venous and arterial phases, the mode of enhancement, and the degree of enhancement were meaningful factors in distinguishing HAS from the common type of gastric cancer. Morphologically, HAS mostly occurs as a solitary tumor. Moreover, its Borrmann classification is usually type III, with a heterogeneous density on plain CT. After a rapid increase in CT attenuation in the arterial phase following enhancement, the CT attenuation differences between the venous and arterial phases become small, with relatively flat curves. In contrast to the progressive intensification in non-HAS tumors, the hepatocellular differentiation zone in HAS is similar to the histologic features of HCC, with abundant blood sinusoids in the interstitial space but with adenocarcinoma components, which is not consistent with the “fast-in, fast-out” intensification of HCC or with the peak intensification in the venous phase of most non-HAS tumors. Therefore, HAS mostly exhibits a continuous type of enhancement. However, in the external validation of data from another center, there were significant differences in six clinical characteristics and CT findings. The different results of the morphological characteristics analysis in the primary and validation cohorts could be explained by the different sample sizes. The primary cohort had a larger sample size and thus might be more representative.

In the HAS group, 72.34% of the patients had pHAS and 27.66% had mHAS. However, there have been no relevant studies on the biological behavior of mHAS. In our study, both the thickest diameter of the tumor and the longest and shortest diameter of the metastatic lymph nodes in mHAS were larger than those in pHAS, and the prognosis was worse in mHAS than in pHAS, indicating that pHAS is an independent risk factor affecting prognosis.

For the treatment of HAS, a combination of radical surgery, chemotherapy, and local interventions is mainly recommended. Moreover, the detection and management of liver metastases should be emphasized during treatment. Roberts et al. (26) reported that 90% of patients with HAS present with lymph node or liver metastases preoperatively. The rate of lymph node metastasis of HAS in this study was 76.60%, and the rate of liver metastasis was 27.66%. The higher liver metastasis rate of HAS was closely related to its high invasiveness to the vasculature (4, 20). Even in the presence of liver metastases, surgical treatment remains the mainstay treatment, and peripheral lymph node dissection is required if the liver metastases can be removed in one stage. The FOLFOX regimen (oxaliplatin + fluorouracil + calcium folinate) may be a potential adjuvant treatment for HAS (27). Liu et al. (13) reported a median survival time of 7.2 months and an overall 5-year survival rate of 20% in patients with HAS. The progression-free survival and overall survival times of patients with HAS in this study were significantly shorter than those of patients with non-HAS tumors. Because of the highly aggressive nature and poor biological behavior of HAS, emphasis should be placed on comprehensive treatment. Fu et al. (25) analyzed the clinicopathologic features and prognosis of 45 patients with HAS and showed that postoperative liver metastases and pathologic TNM stage were independent risk factors affecting prognosis. In this study, we found that the factors affecting the prognosis of patients with HAS were the M stage, overall TNM stage, presence of vascular cancer emboli, and pHAS type, with M stage and pHAS type being the independent risk factors. TNM stage is a prognostic factor for gastric cancer, although our study showed that the M stage has a greater prognostic impact on HAS. Another factor is the pHAS type; however, this is a postoperative pathologic indicator and is not available preoperatively. Further, there are differences in the thickest diameter of the tumors and in the metastatic lymph nodes between simple and mixed tumors, which may help indirectly assess a prognosis.

Our study had some limitations. First, owing to the rarity of HAS, our study was based on a retrospective review, and although validation was conducted using cases from another center, the number of cases was low. Second, as a rare disease, HAS suffers from a lack of understanding of standard diagnosis and therapy, which contributed to the original data’s confusion and imperfection, such as the lack of necessary examinations for clinical diagnosis and the complexity of therapeutic options. Therefore, perspective studies with larger sample size are needed to further analyze CT features of HAS and their association with pathological characteristics and prognosis.

## Conclusion

In conclusion, the clinical features of HAS are atypical and clinicians and radiologists lack familiarity with the features of this tumor. Through a systematic investigation of HAS and comparisons with non-HAS, we found that serum AFP level, M stage, and degree of tumor enhancement on CT are independent factors for differentiating HAS from other gastric cancers. mHAS has a worse prognosis than pHAS, and M stage and pHAS type were independent risk factors affecting the prognosis of patients with HAS. Owing to the aggressive nature and poor prognosis of HAS, rapid clinical intervention and detailed follow-up with CT are essential.

## Data Availability Statement

The original contributions presented in the study are included in the article/**Supplementary Material**. Further inquiries can be directed to the corresponding author.

## Ethics Statement

The institutional review board of The First Affiliated Hospital of Zhengzhou University approved this retrospective study and waived the requirement for informed consent.

## Author Contributions

W-pH and L-mL: research design and writing. C-cL and W-pH: clinical data collection. JL, J-hY, and Y-jH: CT imaging data collection. X-nL and Y-hM: collection and analysis of pathological data. PL and L-mL: CT imaging data analysis. J-bG: manuscript review. All authors contributed to the article and approved the submitted version.

## Funding

This work was supported by the National Natural and Science Fund of China (No. 81971615).

## Conflict of Interest

The authors declare that the research was conducted in the absence of any commercial or financial relationships that could be construed as a potential conflict of interest.

## Publisher’s Note

All claims expressed in this article are solely those of the authors and do not necessarily represent those of their affiliated organizations, or those of the publisher, the editors and the reviewers. Any product that may be evaluated in this article, or claim that may be made by its manufacturer, is not guaranteed or endorsed by the publisher.
