# Supplementary material for: Computed Tomography Features and Clinical Prognostic Characteristics of Hepatoid Adenocarcinoma of the Stomach
Source: Front Oncol. 2021 Dec 9;11:772636. doi: 10.3389/fonc.2021.772636 (PMC8696206; doi:10.3389/fonc.2021.772636)
Supplement: Supplementary file 1 [file DataSheet_1.docx]

***Supplementary Material***

**Table S1** Clinical characteristics and CT findings for validation cohort (n=36)

|  |  | HAS (n=9) | non-HAS (n=27) | *P* value |
| --- | --- | --- | --- | --- |
| Sex | Male | 8 | 22 | 1.000 |
|  | Female | 1 | 5 |  |
| Age (years) |  | 48.33±11.45 | 60.48±10.33 | 0.005^*^ |
| Location | Antrum | 4 | 2 | 0.267 |
|  | Body | 1 | 6 |  |
|  | Cardia and Fundus | 4 | 18 |  |
|  | Involvement of 2 or more sites | 0 | 1 |  |
| Longest diameter of tumor (cm) |  | 7.50(3.75, 10.25) | 4.50(4.00, 6.00) | 0.203 |
| Degree of differentiation | Low | 9 | 16 | 0.034^*^ |
|  | Middle-high | 0 | 11 |  |
| Neural encroachment | Yes | 6 | 24 | 0.032 |
|  | No | 3 | 3 |  |
| Vascular invasion | Yes | 9 | 17 | 0.086 |
|  | No | 0 | 10 |  |
| T stage | 2 | 1 | 3 | 1.000 |
|  | 3 | 4 | 12 |  |
|  | 4 | 4 | 12 |  |
| N stage | 0 | 1 | 5 | 0.685 |
|  | 1 | 1 | 5 |  |
|  | 2 | 2 | 7 |  |
|  | 3 | 5 | 10 |  |
| M stage | 0 | 7 | 26 | 0.148 |
|  | 1 | 2 | 1 |  |
| TNM stage | 1 | 0 | 1 | 0.057 |
|  | 2 | 2 | 6 |  |
|  | 3 | 5 | 20 |  |
|  | 4 | 2 | 0 |  |
| Borrmann type (n=36) | I | 0 | 5 | 0.255 |
|  | II | 3 | 11 |  |
|  | III | 6 | 10 |  |
|  | IV | 0 | 1 |  |
| Thickest diameter (cm) |  | 32.67±10.23 | 14.78±4.43 | 0.001^*^ |
| Plain CT attenuation (HU) |  | 44(41.50, 46.00) | 40.92(29.14, 44.83) | 0.160 |
| Arterial CT attenuation (HU) |  | 82.22±11.48 | 69.70±19.91 | 0.084 |
| Venous CT attenuation (HU) |  | 83.11±13.75 | 82.80±19.67 | 0.966 |
| R_A_ |  | 0.35(0.32, 0.38) | 0.23(0.19, 0.30) | 0.001^*^ |
| R_V_ |  | 0.623±0.14 | 0.57±0.14 | 0.357 |
| HU_A-P_ |  | 38.78±13.98 | 32.05±19.33 | 0.344 |
| HU_V-A_ |  | 0.89±7.73 | 13.10±16.45 | 0.040^*^ |
| Degree of enhancement | Obvious enhancement | 2 | 13 | 0.252 |
|  | Moderate enhancement | 6 | 12 |  |
|  | Mild enhancement | 1 | 2 |  |
| Mode of enhancement | Continuous reinforcement | 5 | 7 | 0.058 |
|  | Progressive reinforcement | 3 | 19 |  |
|  | Ascending and then descending type of reinforcement | 1 | 1 |  |

HAS ＝Hepatoid adenocarcinoma of the stomach; HU＝Hounsfield unit; R_A_＝the ratio of arterial CT attenuation to CT attenuation of the abdominal aorta at the same level; R_V_＝the ratio of venous CT attenuation to CT attenuation of the abdominal aorta at the same level; HU_A-P_＝CT attenuation difference between the arterial phase and plain phase; HU_V-A_＝CT attenuation difference between the venous phase and arterial phase; *. Statistically significant level: P < 0.05

**Table S2** Comparison of clinical pathology information between pHAS and mHAS (n=47)

|  |  | pHAS (n=34) | mHAS (n=13) | *P* value |
| --- | --- | --- | --- | --- |
| Sex | Male | 25 | 8 | 0.654 |
|  | Female | 9 | 5 |  |
| Age (years) |  | 61.65±6.74 | 64.38±18.92 | 0.169 |
| Serum AFP | Elevated | 21 | 8 | 1.000 |
|  | Normal | 13 | 5 |  |
| Serum CA125 | Elevated | 9 | 3 | 1.000 |
|  | Normal | 25 | 10 |  |
| Serum CA724 (n=35) | Elevated | 7 | 4 | 0.329 |
|  | Normal | 19 | 5 |  |
| Serum CA199 | Elevated | 5 | 2 | 1.000 |
|  | Normal | 29 | 11 |  |
| Serum CEA (n=46) | Elevated | 11 | 2 | 0.393 |
|  | Normal | 22 | 11 |  |
| Location | Antrum | 11 | 3 | 0.434 |
|  | Body | 4 | 0 |  |
|  | Cardia and Fundus | 18 | 9 |  |
|  | Involvement of 2 or more sites | 1 | 1 |  |
| Longest short diameter of metastatic lymph node (cm) |  | 1.45(1.00, 2.00) | 1.65(0.78, 3.30) | 0.001^*^ |
| Longest diameter of tumor (cm) |  | 6.39±2.63 | 6.25±4.11 | 0.583 |
| Main symptoms | Abdominal pain / bloating / abdominal discomfort | 19 | 8 | 0.087 |
|  | Acid reflux, heartburn/choking sensation when eating | 7 | 3 |  |
|  | Vomiting of blood/black stool | 5 | 2 |  |
|  | No significant symptoms (physical examination) | 3 | 0 |  |
| Degree of differentiation | Low | 31 | 12 | 0.900 |
|  | Middle-high | 3 | 1 |  |
| Neural encroachment | Yes | 27 | 7 | 0.165 |
|  | No | 7 | 6 |  |
| Vascular invasion | Yes | 25 | 7 | 0.345 |
|  | No | 9 | 6 |  |
| T stage | 1 | 1 | 1 | 0.682 |
|  | 2 | 11 | 3 |  |
|  | 3 | 13 | 4 |  |
|  | 4 | 9 | 5 |  |
| N stage | 0 | 7 | 4 | 0.681 |
|  | 1 | 1 | 1 |  |
|  | 2 | 16 | 4 |  |
|  | 3 | 10 | 4 |  |
| M stage | 0 | 23 | 11 | 0.424 |
|  | 1 | 11 | 2 |  |
| TNM stage | 1 | 2 | 2 | 0.556 |
|  | 2 | 9 | 4 |  |
|  | 3 | 12 | 5 |  |
|  | 4 | 11 | 2 |  |

HAS ＝Hepatoid adenocarcinoma of the stomach; pHAS＝Pure HAS; mHAS＝Mixed HAS; AFP＝alpha-fetoprotein (normal range 0–10 ng/mL); CA＝carbohydrate antigen, CA199 (normal range 0.01–37 U/mL), CA724 (normal range 0–6.9 U/mL), CA125 (normal range 0.01–35 U/mL); CEA＝carcinoembryonic antigen (normal range 0–5 ng/mL); *. Statistically significant level: P < 0.05

**Table** **S3** Comparison of CT features between pHAS and mHAS (n=47)

|  |  | pHAS (n=34) | mHAS (n=13) | *P* value |
| --- | --- | --- | --- | --- |
| Borrmann type | I+II | 6 | 2 | 0.975 |
|  | III | 18 | 7 |  |
|  | IV | 9 | 3 |  |
| Thickest diameter (cm) |  | 2.25±0.83 | 2.90±1.78 | 0.049 |
| Plain CT attenuation (HU) |  | 40.50±6.00 | 42.13±6.85 | 0.764 |
| Arterial CT attenuation (HU) |  | 76.45±17.90 | 81.13±16.97 | 0.056 |
| Venous CT attenuation (HU) |  | 81.55±15.21 | 85.39±11.88 | 0.263 |
| R_A_ |  | 0.29(0.25, 0.33) | 0.25(0.22, 0.35) | 0.118 |
| R_V_ |  | 0.59±0.93 | 0.59±0.08 | 0.846 |
| HU_A-P_ |  | 35.95±17.85 | 39.00±17.22 | 0.081 |
| HU_V-A_ |  | 5.10±14.05 | 4.38±15.34 | 0.256 |
| Degree of enhancement | Obvious enhancement | 11 | 7 | 0.317 |
|  | Moderate enhancement | 16 | 5 |  |
|  | Mild enhancement | 7 | 1 |  |
| Mode of enhancement | Continuous reinforcement | 14 | 5 | 0.789 |
|  | Progressive reinforcement | 15 | 5 |  |
|  | Ascending and then descending type of reinforcement | 5 | 3 |  |

HAS ＝Hepatoid adenocarcinoma of the stomach; pHAS＝Pure HAS; mHAS＝Mixed HAS; HU＝Hounsfield unit; R_A_＝the ratio of arterial CT attenuation to CT attenuation of the abdominal aorta at the same level; R_V_＝the ratio of venous CT attenuation to CT attenuation of the abdominal aorta at the same level; HU_A-P_＝CT attenuation difference between the arterial phase and plain phase; HU_V-A_＝CT attenuation difference between the venous phase and arterial phase; *. Statistically significant level: P < 0.05
